# Predicting brain age for veterans with traumatic brain injuries and healthy controls: an exploratory analysis

**DOI:** 10.3389/fnagi.2025.1472207

**Published:** 2025-05-15

**Authors:** John P. Coetzee, Xiaojian Kang, Victoria Liou-Johnson, Ines Luttenbacher, Srija Seenivasan, Elika Eshghi, Daya Grewal, Siddhi Shah, Frank Hillary, Emily L. Dennis, Maheen M. Adamson

**Affiliations:** ^1^Rehabilitation Service, VA Palo Alto Health Care System, Palo Alto, CA, United States; ^2^Department of Psychiatry and Behavioral Sciences, Stanford Medicine, Stanford, CA, United States; ^3^WOMEN CoE, VA Palo Alto Health Care System, Palo Alto, CA, United States; ^4^Clinical Excellence Research Center, Stanford School of Medicine, Stanford, CA, United States; ^5^Department of Psychology, Palo Alto University, Palo Alto, CA, United States; ^6^Department of Psychology, University of Amsterdam, Amsterdam, Netherlands; ^7^Uniformed Services University of the Health Sciences, Bethesda, MA, United States; ^8^Icahn School of Medicine at Mount Sinai, New York, NY, United States; ^9^Department of Psychology, Pennsylvania State University, University Park, PA, United States; ^10^Department of Neurology, University of Utah School of Medicine, Salt Lake City, UT, United States; ^11^George E. Wahlen Veterans Affairs Medical Center, Salt Lake City, UT, United States; ^12^Department of Neurosurgery, Stanford School of Medicine, Stanford, CA, United States

**Keywords:** traumatic brain injury, chronic health symptoms, aging, structural MRI, brain age

## Abstract

**Background:**

Traumatic brain injury (TBI) is associated with increased dementia risk. This may be driven by underlying biological changes resulting from the injury. Machine learning algorithms can use structural MRIs to give a predicted brain age (pBA). When the estimated age is greater than the chronological age (CA), this is called the brain age gap (BAg). We analyzed this outcome in men and women with and without TBI.

**Objective:**

To determine whether factors that contribute to BAg, as estimated using the brainageR algorithm, differ between men and women who are US military Veterans with and without TBI.

**Methods:**

In an exploratory, hypothesis-generating analysis, we analyzed data from 85 TBI patients and 22 healthy controls (HCs). High-resolution T1W images were processed using FreeSurfer 7.0. pBAs were calculated from T1s. Differences between the two groups were tested using the Mann-Whitney U. Associations between the BAg and other factors were tested using partial Pearson’s *r* within groups, controlling for CA, followed by construction of regression models.

**Results:**

After correcting for multiple comparisons, TBI patients and HCs differed on PCL score (higher for TBI patients) and cortical thickness (CT) in both hemispheres (higher for HCs). Among women TBI patients, BAg was correlated with pBA and hippocampal volume (HV), and among men TBI patients, BAg was correlated with pBA and CT. Among both men and women HCs, BAg was correlated only with CA. Four hierarchical regression models were constructed to predict BAg in each group, which controlled for CA and excluded pBA for multicollinearity. These models showed that HV predicted BAg among women with TBI, while CT predicted BAg among men with TBI, while only CA predicted BAg among HCs.

**Interpretation:**

These results offer tentative support to the view the factors associated with BAg among individuals with TBI differ from factors associated with BAg among HCs, and between men and women. Specifically, BAg among individuals with TBI is predicted by neuroanatomical factors, while among HCs it is predicted only by CA. This may reflect features of the algorithm, an underlying biological process, or both.

## 1 Introduction

### 1.1 Traumatic brain injury

Traumatic brain injury (TBI) is a neurological condition caused by a sudden externally originating injury, resulting in compromised brain function, and which is not caused by a neurodegenerative or neurodevelopmental condition (Roebuck-Spencer and Cernich, 2014). TBI most often results from blunt force trauma to the head, as in the case of falls, athletic injuries, car wrecks, and assaults (including sexual assault and intimate partner violence), while being caused somewhat less often by penetration of object through the skull, as in the case of firearm related suicide (Brasure et al., 2012; Mollayeva et al., 2018). TBIs are categorized as either mild, moderate, or severe. By one common means of classification, mild TBIs involve either no loss of consciousness (LOC), or LOC lasting up to 30 min, while moderate TBIs are those that involve a LOC lasting 30 min–24 h, and severe TBIs involve a LOC lasting > 24 h (Brasure et al., 2012). Mild TBIs are characterized by concussions that are not life-threatening and usually temporary, while severe TBIs may result in unconsciousness, coma, and in the worst cases, death. Among older adults, suffering from a recent TBI with LOC is associated with an increased risk of mortality (Dams-O’Connor et al., 2013). Despite the name, mild TBI, the most common type, is often associated with significant long-term impairments, including measurable cognitive impairment in half of the individuals who suffer this injury, and unemployment in up to a third (McInnes et al., 2017). Worldwide, an estimated 69 million individuals suffer from a TBI annually, with more than 1.7 million Americans being affected each year, contributing to the 5.3 million Americans who suffer from long-term disabilities after TBIs (Thurman et al., 1999; Dewan et al., 2019). TBI is also costly, with an estimated 0.5% (400 billion dollars) of annual global economic output being spent on associated personal and societal costs (McMillan et al., 2011; Maas et al., 2017).

### 1.2 Traumatic brain injury and dementia

There are many factors that increase the risk for dementia, including lack of social interactions (Kuiper et al., 2015), heart disease (Wolters et al., 2018), diabetes (Cheng et al., 2012), and genetic factors [the apolipoprotein E (APOE) ε4 allele] (Chen et al., 2009; Fernández-Calle et al., 2022). Evidence for a significant contribution to dementia risk by TBI is accumulating. A 2003 meta-analysis of 15 case-control studies estimated that individuals who had a TBI severe enough to result in LOC had an approximately 50% increased risk of dementia (Fleminger et al., 2003), and a recent meta-analysis including two-million individuals found that TBI increases the risk of dementia 1.6 times (although this analysis did not address whether risk varies based on TBI severity) (Li et al., 2017). Moreover, acquiring a TBI appears to lower the age of onset of TBI-related neurocognitive syndromes (Mendez, 2017). The risk of dementia increases with a single moderate to severe TBI, and also in the case of repeated mild TBIs (Mendez, 2017).

The neurobiological mechanism linking TBI and dementia appears to involve physical disruptions in white matter tracts and neural networks (Mendez, 2017). TBI can cause axonal injury and induce an inflammatory response in the brain which may persist chronically (Kang et al., 2007). In turn, this may initiate a neurodegenerative cascade, resulting in the development of Alzheimer’s Dementia (AD) or other forms of dementia (Collins et al., 2020). The risk of white matter disruption is present even after a mild TBI, and increases with both the severity and frequency of the injuries (Mendez, 2017).

Clarifying possible links between TBI and dementia is important, given the high economic, societal, and medical burden imposed by dementia on patients, families, and healthcare providers. Dementia is a common disorder affecting more than 55 million people worldwide (World Health Organization [WHO], 2021), including 13.7 million Americans (Parker et al., 2020), with prevalence on the rise (Nichols et al., 2022). Representing a constellation of diseases and disorders, dementia is characterized by a progressive decline in cognitive abilities as well as social and physical functioning (Prince et al., 2013), with the most common types being AD, dementia with Lewy Bodies (DLB), and frontotemporal dementia (FTD) (Alzheimer’s Association, 2014; World Health Organization [WHO], 2021). The specific cognitive domains in which cognitive decline is found depend on the type of dementia, with such decline being more global in AD than in some other forms of dementia (Smits et al., 2015). Annual worldwide economic and societal costs associated with dementia are expected to rise from $818 billion–$2 trillion by 2030 (Parker et al., 2020; World Health Organization [WHO], 2021), representing a 144% increase since 2015.

### 1.3 A machine learning algorithm to predict brain age

In the current study we used a machine learning algorithm, brainageR (Cole, 2018), to predict brain age in Veterans with a history of TBI and identify normative deviations from the typical timeline of age-related brain changes, which may be driven by an injury-related acceleration of underlying aging processes [with the caveat that there is substantial debate as to whether such algorithms can be used to measure or predict longitudinal aging processes (Vidal-Pineiro et al., 2021; Korbmacher et al., 2024c)]. This algorithm has been used to identify such normative deviations from the expected timeline of age-related brain changes among individuals with TBI in other studies (Amgalan et al., 2022; Dennis et al., 2022; Spitz et al., 2022). Here we sought to build on that work and to determine what underlying factors may contribute to such deviations from expected patterns of age-related brain changes. The brainageR algorithm (v2.1) produces an estimate of predicted brain age (pBA) from a raw T1-weighted MRI scan using a Gaussian processes regression, implemented in R, using the kernlab package (Cole, 2018). The model to which the algorithm compares a given T1 was The brainageR model for v2.1 was trained on *n* = 3377 healthy individuals (mean age = 40.6 years, SD = 21.4, age range 18–92 years) from seven publicly available datasets to generate an expected trajectory of normal brain maturation and aging over time, as derived from the structural properties of the brains in the training set (Franke et al., 2010; Cole, 2018). Once these trajectories have been created, the structural properties of brains from an unseen set of test data can be used to place them along those trajectories. Such algorithms have demonstrated accuracy, with measures in adults finding a mean absolute error (MAE) of < 5 years (Cole and Franke, 2017; Cole et al., 2017), which can be measured with high test–retest reliability (intraclass correlation coefficient = 0.90–0.99) (Cole et al., 2017). Although it was once thought that pBAs generated by such algorithms remain stable across the lifespan in healthy populations (Franke et al., 2010), it has been shown that the difference between pBA and chronological age (CA) is negatively correlated with CA (Cole and Franke, 2017), a phenomenon which reflects the training sample’s age bias. In longitudinal data, there is also a tendency for the age bias corrected brain age gap (BAg) to increases at higher ages (Korbmacher et al., 2024c). Estimates of pBA show scan-rescan stability over short periods of time, and, also, show stability across different scanning systems after adjusting for field strength (Franke and Gaser, 2012), although recent work suggests that there may be substantial intraindividual variability at lower field strengths (Korbmacher et al., 2023c). Importantly, age predictions obtained through this algorithmic MRI-based method outperform telomere length, another measure of biological age, for which measurements can be highly variable depending on the extraction and lab analysis practices utilized (Cunningham et al., 2013; Sanders and Newman, 2013; Martin-Ruiz et al., 2015; Franke and Gaser, 2019). It is worth noting, however, that when an individual’s brain anatomy is considered deviant by the model, that deviation is reflected in repeated brain age estimates, independent of pathology (Korbmacher et al., 2023c). Additionally, although there exist algorithms that may demonstrate even higher accuracy than brainageR (More et al., 2023; Korbmacher et al., 2024d), for the current analysis we chose to use brainageR because it has been used in other studies to examine brain age in the context of TBI (Amgalan et al., 2022; Dennis et al., 2022; Dennis et al., 2024). While there is some evidence of model-dependent results in brain age estimations, a cross-model comparison is beyond the scope of the current work but should be undertaken in a future study (More et al., 2023; Korbmacher et al., 2024d). Additional sources of variability that may impact the accuracy of algorithmic brain age predictions include feature sets (multimodal *vs.* unimodal) (Rokicki et al., 2021; Jirsaraie et al., 2023; Korbmacher et al., 2023a), and software version (Korbmacher et al., 2024d). All things considered, it is a tool with substantial potential but which is not without error and which must be applied with care.

The difference or gap between pBA and CA, called here the BAg, may be able to serve as a biomarker of altered aging processes in the brain. This gap may be driven by the presence of anatomical features such as cortical thickness that are more typical of advanced age than the individual’s current age. In a large study of 73-year-olds, for every additional year that an individual’s pBA was older than their CA, there was a 6% increased risk of death (Cole et al., 2018). The same study also found associations between pBA and lower grip strength, lower forced expiratory volume, slower walking time, and a composite measure of fluid cognition. There can be many factors contributing to this change in BAg. Being a long-term meditator (Luders et al., 2016) or a trained musician (Rogenmoser et al., 2018) appears to reduce pBA, relative to CA, resulting in a negative BAg. On the other hand, being born extremely preterm (prior to the 27th week of gestation) (Hedderich et al., 2022), having schizophrenia (Nenadić et al., 2017), being obese (Kolenic et al., 2018), or having diabetes (Franke et al., 2013) are associated with having a pBA that is greater than one’s CA, or a positive BAg. Other covariates of pBA and/or BAg include waist-to-hip ratio, diabetes, hypertension, smoking, matrix puzzles solving, and job and health satisfaction (Korbmacher et al., 2023b).

Algorithmically measured brain age may be able to serve as a predictor of dementia. People diagnosed with Alzheimer’s have been shown to have greater apparent BAg in neuroimaging data (Franke and Gaser, 2012). Also, in people with mild cognitive impairment (MCI), pBA was a significant predictor of progression to dementia within 3 years of the MRI to which the algorithm was applied (Gaser et al., 2013). This may indicate that pBA can be sensitive to subtle changes in the brain that occur before overt disease manifestation, although the current sensitivity of pBA using existing methods is a matter of some debate (Kaufmann et al., 2019; Korbmacher et al., 2023c; Tetereva and Pat, 2023; Korbmacher et al., 2024c; Wang et al., 2024). In progressive neurodegenerative conditions, tools that identify individuals at increased risk of future disease onset could be particularly useful, both for clinical practice and for the design of clinical trials (Cole et al., 2019).

TBI appears to produce deviations from the expected pattern of age-related anatomical brain changes, such that these individuals tend to display greater BAg (Franke and Gaser, 2012; Cole et al., 2015). MRI-derived estimates of gray matter and white matter volume indicate that TBI can produce accelerated atrophy to a degree which is atypical for normal aging (Cole et al., 2015). There have, so far, been only a few studies examining the relationship between TBI and age-related brain changes (Franke and Gaser, 2012; Cole et al., 2015; Amgalan et al., 2022). Given the heightened risk of dementia for individuals with TBI, and the possibility of predicting dementia onset using brain age prediction, it is important to determine what characteristics may contribute to the brain age predictions generated by these algorithms in individuals with TBI.

### 1.4 Purpose of this study

In the current study, we sought to test a few distinct hypotheses. Our first hypothesis was that participants with TBI would show a larger positive BAg, relative to HCs. Then, we sought to whether the factors associated with the BAg, using a Pearson’s correlation, would differ between participants with TBI and HCs, with our hypothesis being that they would. Finally, we sought to construct a regression model that would most accurately predict the BAg for each group using the available data, in order to determine whether the factors predictive of BAg would differ between groups, with our hypothesis being that they would. In the process of conducting the analysis, we also tested for sex differences in an exploratory manner.

### 1.5 Relevance

We hope that this study can add to the growing body of literature on the relationship between TBI and algorithmically modeled BAg. We further hope that can help to clarify the demographic and biological correlates of that relationship. Our study was conducted among US Veterans, a population that is especially impacted by TBI.

## 2 Materials and methods

### 2.1 Participants

For this study, 23 HCs (11 women), and 94 participants with mild (*n* = 61), moderate (*n* = 17), or severe (*n* = 16) TBI (27 women), were recruited. The majority of participants with TBI were recruited either from the Santa Clara Valley Medical Center, Rehabilitation Research Center, or the VA Palo Alto Health Care System (VAPAHCS). Others answered advertisements posted at Stanford University and communities throughout Santa Clara County, California. HCs were recruited through advertisements and among colleagues. All participants underwent clinical interviews with a neurologist, psychiatrist, or physical medicine and rehabilitation specialist to gather information about their TBI history and chronic symptoms after injury. TBI severity was measured using the Ohio State University Traumatic Brain Injury Identification Method (OSU TBI-ID). Regarding inclusion criteria, all participants had to be US Veterans, be capable of safely undergoing an MRI, and not have another neurological condition that explain morphological brain changes better than TBI. TBI patients had to qualify for a TBI diagnosis using the OSU TBI-ID. All participants provided informed consent according to the Declaration of Helsinki. The experimental protocol was approved by the Institutional Review Board of Stanford University and by the VAPAHCS Scientific Review Board. Nine individuals were excluded for being > 3 standard deviations from the mean on either pBA or on one of the structural brain elements being measured. One individual with brain age > 20 years was not > 3 standard deviations (SD) from the mean, but upon inspection was found to have overdrawn cerebrospinal fluid (CSF) masks, including meninges and frontal sinus. Of these ten exclusions one was HC and nine were TBI, leaving 85 TBI patients and 22 HCs (see Table 1 for sample characteristics). We chose to use Veterans rather than Active-Duty Service Members, because we were interested in the long term chronic effects of TBI on brain anatomy and brain age, rather than the acute effects. For the same reason, we did not enroll participants who were recently returned from a war zone.

**TABLE 1:** Demographics and other participant characteristics for patients and HCs.

TBI patients	*N*	Minimum	Maximum	Mean	SD	
CA (years)	85	20.00	76.81	42.65	13.39	
Education (years)	83	11.00	21.00	15.33	2.23	
PCL score	64	0.00	70.00	38.44	17.03	
Age at injury (years)	75	2.00	60.00	25.89	13.38	
Time since injury (years)	75	0.08	64.25	17.09	15.23	
pBA (years)	85	17.77	80.77	43.99	15.44	
Left CT (mm)	85	1.95	2.41	2.20	0.09	
Right CT (mm)	85	1.91	2.41	2.19	0.09	
CT (mm)	85	1.93	2.41	2.19	0.09	
Left HV (mm^3^)	85	2,988.75	5,477.01	4,111.81	462.77	
R HV (mm^3^)	85	3,069.09	5,393.64	4,227.74	495.49	
HV (mm^3^)	85	3,071.48	5,435.32	4,169.78	459.01	
TICV (mm^3^)	85	1,156,049.20	1,845,470.68	1,517,952.52	143,646.14	
BAg (years)	85	–21.87	32.67	1.34	8.15	
	**M**	**F**	**Other**		**Yes**	**No**
Sex	62	23	0	PTSD dx	46	39
	1	2	3			
TBI severity	57	16	12			
**Healthy controls**	***N***	**Minimum**	**Maximum**	**Mean**	**SD**	
CA (years)	22	23.00	54.00	39.05	10.83	
Education (years)	22	12.00	26.00	17.00	3.24	
PCL score	13	17.00	61.00	26.69	12.01	
pBA (years)	22	24.44	57.89	39.76	9.08	
Left CT (mm)	22	2.10	2.46	2.28	0.09	
Right CT (mm)	22	2.08	2.40	2.25	0.08	
CT (mm)	22	2.09	2.43	2.26	0.08	
Left HV (mm^3^)	22	3,655.89	5,091.55	4,235.58	367.88	
R HV (mm^3^)	22	3,677.16	5,665.87	4,348.19	448.28	
HV (mm^3^)	22	3,713.31	5,378.71	4,291.89	393.44	
TICV (mm^3^)	22	1,192,109.01	1,695,683.90	1,435,432.82	139,217.15	
BAg (years)	22	–16.62	14.92	0.72	7.09	
	**M**	**F**	**Other**		**Yes**	**No**
Sex	11	11	0	PTSD dx	2	11

### 2.2 MR image acquisition

MRI imaging data was acquired at VAPAHCS on a GE 3T Discovery MR750 scanner with an 8-channel head coil (GE Medical Systems, Milwaukee, WI). Each MRI session lasted approximately 1 h and included T1 and T2-weighted imaging (T1W & T2W), diffusion weighted imaging (DWI), resting state (T2*), susceptibility weighted imaging (SWI), and fluid attenuation inversion recovery (FLAIR). Only high-resolution T1W images were used for the current analyses. The T1W sequence used a three-dimensional spoiled-gradient recalled acquisition (3D-fast spoiled gradient echo MRI) in steady state with the parameters: TR = 7.3 ms; TE = 3.0 ms; flip angle = 11 degrees; 272 axial slices with the slice thickness = 1.2 mm with 0.6 between slices; field of view = 250 mm; voxel dimensions: 1.05 × 1.05 × 0.60 mm.

### 2.3 Anatomical image preprocessing

High resolution T1W anatomical images were processed using FreeSurfer 7.0, which includes intensity normalization (Sled et al., 1998), segmentation (Fischl et al., 2004), inflation of surfaces to spheres (Fischl et al., 1999a), and spherical registration of spherical surfaces to a standard template (Fischl et al., 1999b). FreeSurfer provides cortical thickness and neuroanatomical parcellation of the cortex and subcortical structures for all subjects (Desikan et al., 2006). The cortical thickness (Adamson et al., 2020) and volume of hippocampus (Ertekin et al., 2013) were collected as imaging markers for brain age prediction after they were normalized for inter-subject variation in brain size, defined and measured as total intracranial volume (TICV) (Schwarz et al., 2016).

### 2.4 Calculation of predicted brain age from anatomical images

**FIGURE 1:**
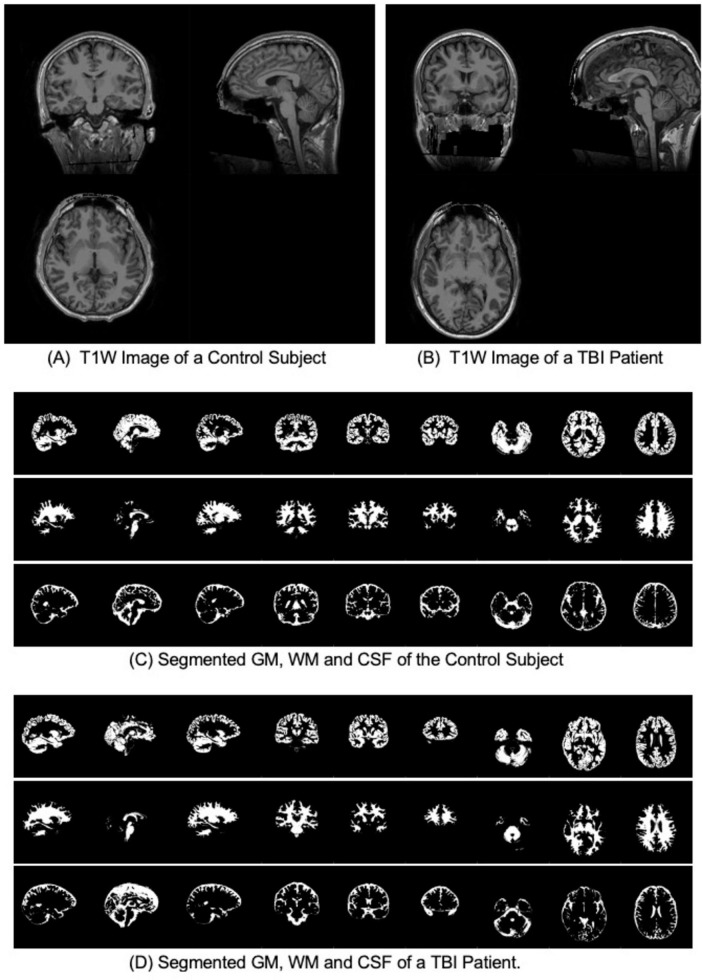
T1W and segmented gray matter (GM), white matter (WM) and Cerebrospinal fluid (CSF) for a control subject and a TBI patient with the same CA of 27 years old. **(A)** T1W Image of a Control Subject. **(B)** T1W Image of a TBI Patient. **(C)** Segmented GM, WM and CSF of the Control Subject. **(D)** Segmented GM, WM and CSF of a TBI Patient.

pBAs were calculated from the T1W MR images using brainageR pre-trained models (Cole, 2018). The software includes the following steps: (1) The raw T1W MRI images were preprocessed using SPM12 (Ashburner et al., 2014) for segmentation into gray matter (GM), white matter (WM) and corticospinal fluid (CSF) maps, then normalized to Montreal Neurological Institute (MNI) space with a 4 mm smoothing kernel; (2) machine learning analysis was conducted using the Pattern Recognition for Neuroimaging Toolbox (PRoNTo) (Schrouff et al., 2013) and run on GM and WM separately; (3) model validation was conducted to ensure independence between training and test sets and to enable an unbiased demonstration of model generalizability; (4) Principal Components Analysis (PCA) was applied to predict an age value with the trained model (Karatzoglou et al., 2004). Values for pBA were obtained from the machine learning analysis of neuroimaging data for all HCs and TBI patients. See Figure 1 for examples of T1s from a HC and TBI patient, respectively.

### 2.5 Statistical analyses

**FIGURE 2:**
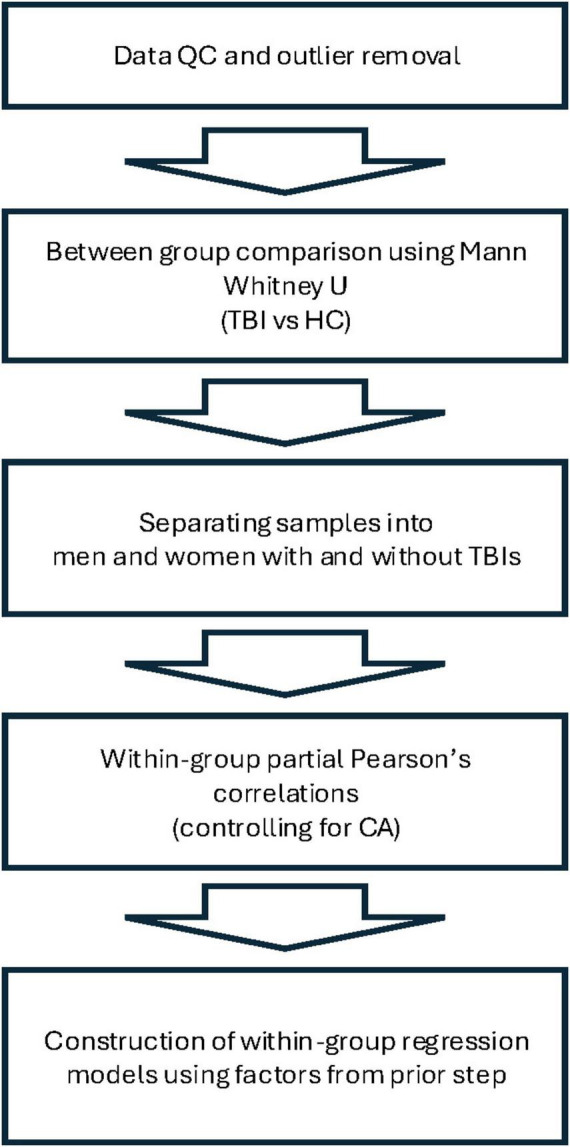
Overview of analysis.

In this exploratory hypothesis-generating analysis, we compared TBI participants and HCs on several biological and demographic parameters. Mann-Whitney U tests were conducted to test for pairwise comparisons between TBI patients and HCs in the following variables: CA, years of education, score on the Post-traumatic Stress Disorder Checklist for DSM-5 (PCL-5), pBA, BAg, overall cortical thickness (CT), left hemisphere CT, right hemisphere CT, overall hippocampal volume (HV), left hemisphere HV, right hemisphere HV, and total intracranial volume (TICV). We then separated each group by sex and conducted Pearson’s correlations within each subgroup in order to identify factors that were associated with BAg. In accordance with recommendations from the creator of the brainageR algorithm, we controlled for CA in these correlations by conducting a partial Pearson’s r with CA as the controlled for variable (this also normalized the underlying variables, obviating the need for a nonparametric test). Factors considered as potential correlates for BAg among TBI patients included years of education, PCL score, age of injury, time since injury (years), pBA, overall CT, and overall HV. For HCs, factors considered included years of education, PCL score, pBA, overall CT, and overall HV. The factors identified (pBA and HV for women with TBIs, pBA and CT for men with TBIs, and pBA and CA for men and women HCs) were then used to construct four multiple regression models for BAg within each subgroup, and CA was included as a variable in all models, as advised by James Cole. All statistical tests were conducted using IBM SPSS Statistics for Macintosh, Version 29. Anatomical volumes were corrected for TICV during Freesurfer analysis. An FDR correction was conducted to control for multiple comparisons during each stage of the analysis. See Figure 2 for an overview of the analysis.

## 3 Results

### 3.1 Between-group comparisons

Summaries of demographic and biological parameters for each group can be seen in Table 1. The results showed that the groups differed significantly on the following measures (note that both uncorrected and FDR corrected *p*-values are shown): compared to TBI participants, HCs had more years of education [TBI *M*: 15.33 (*SD*: 2.23) *vs.* HC *M*: 17.00 (*SD*: 3.24), *p* = 0.026, pFDR = 0.056], greater overall cortical thickness [TBI *M*: 2.19 (*SD*: 0.09) *vs.* HC *M*: 2.26 (*SD*: 0.08), *p* = 0.002, pFDR = 0.012, in mm], and greater cortical thickness in both the left [TBI *M*: 2.20 (*SD*: 0.09) *vs.* HC *M*: 2.28 (*SD*: 0.09), *p* = 0.0008, pFDR = 0.009, in mm] and right [TBI *M*: 2.19 (*SD*: 0.09) *vs.* HC *M*: 2.25 (*SD*: 0.08), *p* = 0.003, pFDR = 0.012, in mm] hemispheres. Compared to HCs, TBI participants had higher scores on the Post-traumatic Stress Disorder Checklist for DSM-5 (PCL-5) (Blevins et al., 2015) [TBI *M*: 38.70 (*SD*: 17.08) *vs.* HC *M*: 26.69 (*SD*: 12.01), *p* = 0.011, pFDR = 0.033], as well as greater TICV [TBI *M*: 1,517,952.52 (*SD*: 143,646.14) *vs.* HC *M*: 1,435,432.82 (*SD*: 393.44), *p* = 0.028, pFDR = 0.056, in mm^3^]. The two groups did not differ significantly regarding pBA, BAg, or CA, despite participants with TBI being numerically higher on all three compared to HCs. This differs from some other studies (Ertekin et al., 2013; Cole et al., 2017), and may be a consequence of our relatively small HC sample. Results shown in Table 2. All uncorrected significant comparisons survived an FDR correction (Benjamini and Hochberg, 1995) (FDR crit. value = 0.05) except for TICV and education, as described above.

**TABLE 2:** Nonparametric pairwise comparisons using the Mann-Whitney U.

Factor	TBI patients *M* (SD)	HCs *M* (SD)	Test statistic	p uncorr.	pFDR
Education (years)	15.33 (2.23)	17.00 (3.24)	634.00	0.026	0.056
	*n* = 83	*n* = 22			
PCL-5 (points)	38.44 (17.03)	26.69 (3.33)	229.50	0.011*	0.033
	*n* = 64	*n* = 13			
Cortical thickness (mm)	2.19 (0.09)	2.26 (0.08)	527.00	0.002*	0.012
	*n* = 85	*n* = 22			
LH cortical thickness (mm)	2.20 (0.09)	2.28 (0.09)	499.00	<0.001*	0.009
	*n* = 85	*n* = 22			
RH corticol thickness (mm)	2.19 (0.09	2.25 (0.08)	554.50	0.003*	0.012
	*n* = 85	*n* = 22			
TICV (in mm^3^)	1,517,952.52 (143,646.14)	1,435,432.82 (139,217.15)	650.00	0.028	0.056
	*n* = 85	*n* = 22			

Only significant differences are shown. Comparisons that survived an FDR correction are marked with a *.

### 3.2 Correlations with brain age gap

A partial Pearson’s *r*, controlling for CA, was used to identify factors correlated with BAg, for the purpose of identifying candidates for subsequent regression models within each group (TBI and HCs) with men and women considered separately. Factors considered as correlates for BAg among TBI patients included years of education, PCL score, age of injury, time since injury (years), pBA, overall CT, and overall HV. For HCs, factors considered included years of education, PCL score, pBA, overall CT, and overall HV. Results are shown in Table 3. Both uncorrected and FDR corrected *p*-values are given in the table, and below.

**TABLE 3:** Factors correlated with BAg among TBI patients and HCs, while controlling for CA.

	Pearson’s r	p uncorrr.	pFDR
**Women TBI patients**			
pBA	1.0	<0.001*	<0.007
HV	–0.567	0.006*	0.021
**Men TBI patients**			
pBA	1.0	<0.001*	<0.0007
CT	–0.370	0.003*	0.011
HV	–0.289	0.024	0.056
**Women HCs**			
pBA	1.0	<0.001*	<0.005
CT	–0.685	0.029	0.073
**Men HCs**			
pBA	1.0	<0.001*	<0.005
Years education	–0.633	0.049	0.123

Only significant results shown. All tests 2-tailed. Results that survive an FDR correction are marked with a *.

Among women with TBIs, factors that were significantly correlated with BAg included: pBA (*r* = 1.0, *p* = < 0.001, df = 20, pFDR = < 0.007), and HV (*r* = –0.567, *p* = 0.006, df = 20, pFDR = 0.021), both of which survived FDR correction. Among men with TBIs, factors that were significantly correlated with BAg included: pBA (*r* = 1.00, *p* < 0.001, df = 59, pFDR = 0.0007), CT (r = –0.370, *p* = 0.003, df = 59, pFDR = 0.011), and HV (*r* = –0.289, *p* = 0.024, df = 59, pFDR = 0.056). Only pBA and CT survived an FDR correction.

Among women who were HCs, factors that were significantly correlated with BAg included: pBA (*r* = 1.00, *p* = < 0.001, df = 8, pFDR < 0.005), and CT (*r* = –0.685, *p* = 0.029, df = 8, pFDR = 0.073), with only pBA surviving an FDR correction. Among men who were HCs, factors that were significantly correlated with BAg included: pBA (*r* = 1.00, *p* < 0.001, df = 8, pFDR < 0.005), and years of education (*r* = –0.633, *p* = 0.049, df = 8, pFDR = 0.123), with only pBA surviving an FDR correction.

It is worth noting that in all 4 groups above a perfect partial correlation was observed between pBA and BAg after controlling for CA, suggesting collinearity between the two variables. This being the case, caution will be used in incorporating these variables into subsequent regression models.

Separately, a Spearman’s rho was used to test for a relationship between TBI severity among men and women with TBIs. Results were nonsignificant for both populations.

### 3.3 Regression models to predict brain age gap

Four regression models were constructed, one each for women with TBIs, men with TBIs, women who were HCs, and men who were HCs. All four made use of variables identified during the previously reported partial correlations.

**FIGURE 3:**
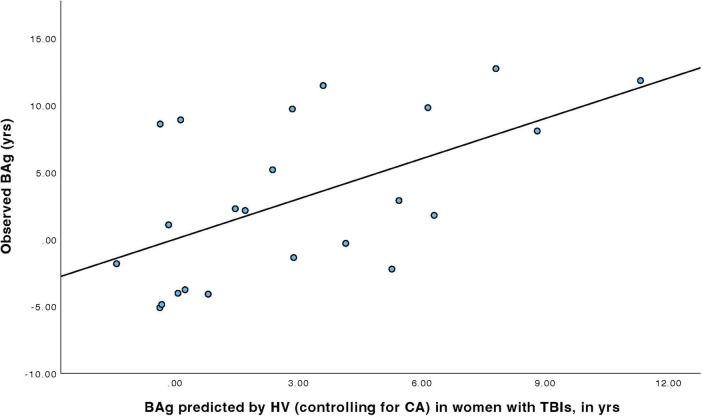
Regression model predicting BAg among women with TBI using HV, while controlling for CA.

For women with TBIs (Table 4), a linear regression model was constructed to predict BAg using the predictors: CA (included as a control), pBA, and HV (all identified during the earlier Pearson’s correlations). By examining collinearity statistics (i.e., Durbin Watson, tolerance, variance inflation factor (VIF), and collinearity index) it was determined that pBA was creating excessive collinearity, and so it was removed from the model. The subsequent model (Model 1) included as predictors: CA and HV, and was judged to be free of collinearity. Other statistical assumptions were also checked and judged to have been adequately met. The overall regression model was statistically significant, F(2, 20) = 4.79, *p* = 0.020, pFDR = 0.023, and accounted for approximately 32.4% of the variance in BAg (*R*^2^ = 0.324, adjusted R^2^ = 0.256). The regression coefficient for HV was significant, B = –0.009, SE = 0.003, β = –0.566, t(20) = –3.08, *p* = 0.006, pFDR = 0.018, indicating that higher HV was associated with a lower BAg. In contrast, CA, included as a control, was not a significant predictor of BAg, B = –0.045, SE = 0.089, β = –0.094, t(20) = –0.508, *p* = 0.617, pFDR = 0.617 (Figure 3).

**TABLE 4:** Predicting BAg among women TBI patients using HV while controlling for CA.

*R*	*R* ^2^	*Adj. R^2^*	SE			
0.569	0.324	0.256	5.177			
**ANOVA**	**SS**	**df**	**MS**	***F***	**p uncorr.**	**pFDR**
Regr.	256.06	2	128.303	4.788	0.020*	0.023
Res.	535.934	20	26.797			
Total	792.540	22				
Coefficients	Unst.		St.			
	***B***	**SE**	**Beta**	***t***	**p uncorr.**	**pFDR**
Constant	42.722	13.160		3.246	0.004	
CA	–0.045	0.089	–0.094	–0.508	0.617	0.617
HV	–0.009	0.003	–0.566	–3.076	0.006*	0.018

Significant results that survive an FDR correction are marked with a *.

**FIGURE 4:**
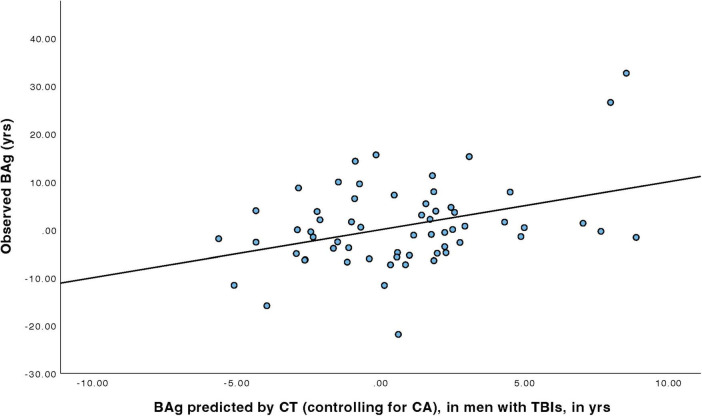
Regression model predicting BAg among men with TBI using CT, while controlling for CA.

For men with TBIs (Table 5), a linear regression model was constructed to predict BAg using CA, pBA, and cortical thickness. By examining collinearity statistics it was determined that pBA was creating excessive collinearity, and was removed. The subsequent model (Model 2) included as predictors CA (as a control) and CT, and was judged to be free of collinearity. Other assumptions were also checked and judged to have been adequately met. The overall model was statistically significant, F(2, 59) = 4.76, *p* = 0.012, pFDR = 0.023, and accounted for approximately 13.9% of the variance in BAg (R^2^ = 0.139, adjusted R^2^ = 0.110). CT was a significant predictor of BAg, B = –42.324, SE = 13.857, β = –0.442, t(59) = –3.05, *p* = 0.003, pFDR = 0.018, suggesting that greater CT was associated with a lower BAg. CA was also a significant predictor (uncorrected), B = –0.190, SE = 0.093, β = –0.295, t(59) = –2.04, *p* = 0.046, pFDR = 0.0552, although it did not survive an FDR correction (Figure 4).

**TABLE 5:** Predicting BAg among men TBI patients using CT while controlling for CA.

*R*	*R* ^2^	Adj. *R*^2^	SE			
0.373	0.139	0.110	8.287			
**ANOVA**	**SS**	**df**	**MS**	***F***	**p uncorr.**	**pFDR**
Regr.	653.253	2	326.626	4.756	0.012*	0.023
Res.	4052.108	59	68.680			
Total	4705.361	61				
Coefficients	Unst.		St.			
	***B***	**SE**	***Beta***	***t***	***p uncorr.***	***pFDR***
Constant	101.706	32.784		3.102	0.003	
CA	–0.190	0.093	–0.295	–2.040	0.046	0.0552
CT	–42.324	13.857	–0.442	–3.054	0.003*	0.018

Significant results that survive an FDR correction are marked with a *.

**FIGURE 5:**
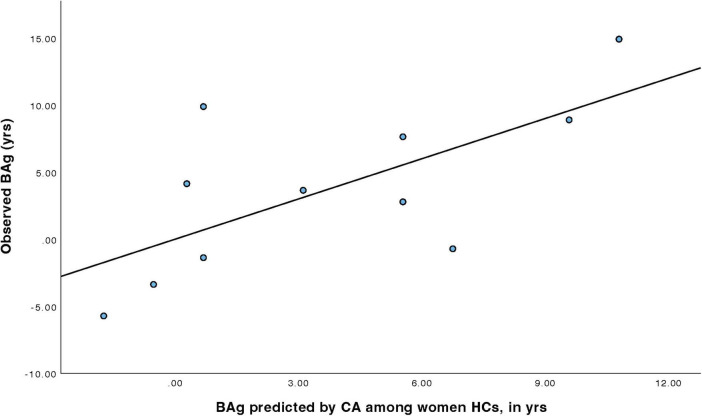
Regression model predicting BAg among women HCs using CA.

For women HCs (Table 6), a model was constructed to predict BAg using CA and pBA. By examining collinearity statistics it was determined that pBA was creating excessive collinearity, and was removed, leaving only CA. This model (Model 3) was statistically significant, F(1, 9) = 7.54, *p* = 0.023, pFDR = 0.023, explaining approximately 45.6% of the variance in BAg (R^2^ = 0.456, adjusted R^2^ = 0.395). CA was a significant negative predictor of BAg, B = –0.404, SE = 0.147, β = –0.675, t(9) = –2.75, *p* = 0.023, pFDR = 0.035, indicating that higher CA was associated with a lower BAg (Figure 5).

**TABLE 6:** Predicting BAg among women HCs using CA as a predictor.

*R*	*R* ^2^	Adj. *R*^2^	SE			
0.675	0.456	0.395	4.87			
**ANOVA**	**SS**	**df**	**MS**	***F***	**p uncorr.**	**pFDR**
Regr.	178.505	1	178.505	7.537	0.023*	0.023
Res.	213.161	9	23.685			
Total	391.666	10				
Coefficients	Unst.		St.			
	***B***	**SE**	**Beta**	***t***	**p uncorr.**	**pFDR**
Constant	20.093	6.147		3.269	0.010	
CA	–0.404	0.147	–0.675	–2.745	0.023*	0.035

Significant results that survive an FDR correction are marked with a *.

**FIGURE 6:**
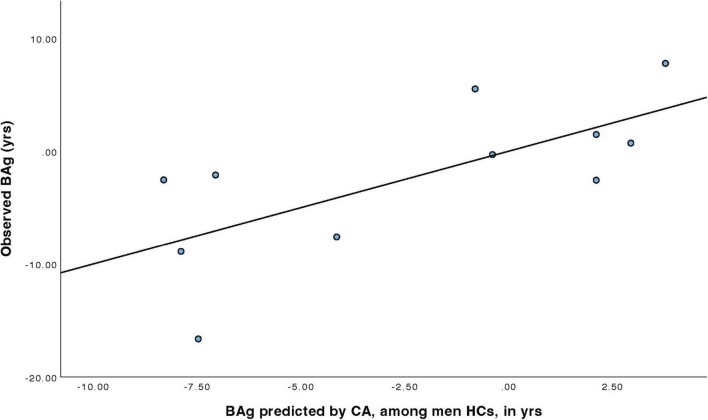
Regression model predicting BAg among men HCs using CA.

For men HCs (Table 7), a model was constructed to predict BAg using CA and pBA. By examining collinearity statistics it was determined that pBA was creating excessive collinearity, and was removed, leaving only CA. This model (Model 4) was statistically significant, F(1, 9) = 8.68, *p* = 0.016, pFDR = 0.023, explaining approximately 49.1% of the variance in BAg (R^2^ = 0.491, adjusted R^2^ = 0.434). CA was a significant negative predictor of BAg, B = –0.417, SE = 0.141, β = –0.701, t(9) = –2.95, *p* = 0.016, pFDR = 0.032, indicating that higher CA was associated with a lower BAg (Figure 6).

**TABLE 7:** Predicting BAg among men HCs using CA as a predictor.

*R*	*R* ^2^	Adj. *R*^2^	SE			
0.701	0.491	0.434	5.141			
**ANOVA**	**SS**	**df**	**MS**	***F***	**p uncorr.**	**pFDR**
Regr.	229.355	1	229.355	8.679	0.016*	0.023
Res.	237.844	9	26.427			
Total	467.199	10				
Coefficients	Unst.		St.			
	***B***	**SE**	**Beta**	***t***	**p uncorr.**	**pFDR**
Constant	13.371	5.533		2.417	0.039	
CA	–0.417	0.141	–0.701	–2.946	0.016*	0.032

Significant results that survive an FDR correction are marked with a *.

We note here that all models survived an FDR correction, but that there is assumed to be some inflation of the model fit metrics resulting from the inclusion of CA as a control. We also note the predictor coefficients were subjected to FDR correction across models, not merely within models. For all FDR corrections the significance criterion was set at 0.05.

### 3.4 Transparency, rigor, and reproducibility summary

Sample began with 94 TBI patients and 23 healthy controls. Nine individuals were excluded for being > 3 standard deviations from the mean on either pBA or on one of the structural brain elements being measured. One individual with brain age > 20 years was not > 3 SD from the mean, but upon inspection was found to have overdrawn CSF masks, including meninges and frontal sinus. Of these ten exclusions one was HC and nine were TBI, leaving 85 TBI patients and 22 HCs. Hypotheses were not preregistered because this study was considered to be exploratory in nature, and we intend that it will provide the basis for future replication attempts. We had a power of 52.5% to detect differences between the means of the two groups using the Mann-Whitney U, assuming a moderate effect size of d = 0.5. When performing the correlations, we had a power of 99.95% to detect a moderate correlation (= 0.5) within the TBI group, and a power of 73.11% to detect an equivalent correlation within the healthy control group. Linear regression models conducted amongst TBI patients were powered at the 89.1% level to detect a moderate effect size, while among healthy controls the models were powered at the 30.4% level. Limitations of this exploratory study, such as the small number of healthy controls, are noted in the text. All power calculations conducted with G*Power. Because of the relatively low power available for Mann-Whitney U above, a weakness of our own study, we recommend that future attempts at replication include at least *n* = 70 in each group, which will give power > 80% for effect size d = 0.5.

## 4 Discussion

In the current study, we sought to use the brainageR algorithm to examine whether TBI patients showed evidence of larger positive BAg relative to HCs who had never experienced a TBI, and, to determine whether factors associated with the BAg differed between participants with TBI and those who had never had one, and, finally, to determine whether the factors that predicted the BAg would also differ between the two groups, as well as between subgroups. In so doing, we sought to determine not only whether the two groups differed in regards to their BAg, but also whether the underlying pattern of changes that were associated with that outcome differed between them.

First, contrary to our hypothesis that individuals with TBI would show either larger pBAs or BAgs relative to HCs, our TBI participants did not significantly differ from HCs with regard to either pBA or BAg (Table 2). This lack of difference between groups in pBA and BAg should be interpreted carefully. First, as noted above, our sample had relatively low power to detect this difference. Also, there is some evidence that algorithmically measured BAg is not especially sensitive to aging (Vidal-Pineiro et al., 2021; Korbmacher et al., 2024c), and it appears to be affected by a variety of other factors (Korbmacher et al., 2023b), which were not controlled for in this comparison. However, our TBI participants did exhibit significantly reduced cortical thickness, both overall and in each hemisphere, relative to HCs (Thambisetty et al., 2010). The lack of a difference in pBA and BAg between the TBI and HC groups was surprising given the literature indicating that TBI tends to be associated with greater BAg, relative to individuals without TBIs (Dennis et al., 2022).

Second, we predicted that the factors associated with BAg, as revealed by a partial Pearson’s *r* controlling for CA, would differ between persons with TBI and HCs, and such differences were apparent (Table 3). We found that BAg significantly associated with pBA among all participants, however, BAg was only associated with HV among women with TBIs, and was only associated with CT among men with TBIs.

Third, we sought to test whether the factors that were associated with BAg and which were predictive of its magnitude in linear regression models would differ between patients with TBIs and HC, and such differences were apparent. These regression models were constructed using these factors to predict BAg in each population, with CA included as a covariate. pBA was excluded from this stage of analysis because of problems with multicollinearity. In these models, among women with TBIs, higher HV was associated with a lower BAg, while among men with TBIs greater CT and was associated with a lower BAg. Among both men and women HCs, higher CA predicted a smaller BAg. The adjusted *R*^2^ for these models ranged from 0.110 (CT among men with TBI) to 0.434 (CA among men HCs).

The interpretation of these is challenging. The analysis produced by the brainageR algorithm does not provide any neuroanatomical specificity with regard to which features are used in generating the pBA. There are algorithmic brain age prediction models which attempt to deliver explainable predictions, but with brainageR it is the case that no feature importance rankings or any other explainable factors used in the analytic process are obtainable (at least not with the version we used), making it a black box. Additionally, there are technical challenges to making explicit the processes by which the Gaussian process regressions produce their output (Franke and Gaser, 2019). A tentative potential explanation for the differences seen here between participants with TBI and HCs is that while the anatomical brain changes that occur during normal aging might be characterized as global and diffuse, those which occur in individuals with a TBI might be more focused on specific structures affected by their injury. There is evidence from several large scale studies that brain regions near the ventricles might be more sensitive to aging (Fujita et al., 2023; Korbmacher et al., 2024a), also found when looking at age-associations and brain age (Leonardsen et al., 2022; Korbmacher et al., 2023a; Korbmacher et al., 2024b), a finding that may run against the suggestion above that are more global and diffuse in HC brains compared to those of TBI patients, but which may support the contribution of HV to greater BAg among women with TBIs described here.

Next, we hypothesized that the ability to predict BAg using multiple regression models constructed from the previously identified factors would differ between persons with TBI and HCs; and our hypothesis was supported.

In summary, we found that in our sample the factors which predicted BAg differed between TBI patients and HCs, as well as between men and women with TBIs. For women with TBIs, BAg was most strongly predicted by HV, while for men with TBIs, it was most strongly predicted by CT. For both men and women in the HC group, BAg was best predicted by CA. We speculate that sustaining a TBI alters the underlying network of causal factors which contribute to age-related neuroanatomical brain changes, such that age-related brain changes are more driven by the injury, while among HCs the determinants of age-related brain changes appear less related to discrete anatomical features of the brain, but may instead exert their impacts in more subtle ways. However, clarifying the nature of this relationship will require larger sample sizes and more sophisticated age prediction algorithms.

Limitations to our study included a relatively small number of HCs, relative to the number of TBI patients. This may impose limits on the statistical conclusions that can be drawn. Future studies of this sort should include larger well-matched HC samples (at least 70 per sample, as mentioned above). Future undertakings of this kind of work should also include more women in the patient group. Another limitation was that we did not have access to detailed medical histories, and thus could not evaluate the role that other health factors may have played in age-related brain change differences (or the lack thereof) between TBI participants and HCs. Additionally, there was a lack of other biomarkers for correlations, such as genetic or proteomic measures, which have been correlated with prediction of AD (Bai et al., 2021). Lastly, neuroimaging was based upon one time point, so it is difficult to state that these findings are the result of a progressive process. We emphasize here the exploratory nature of the current analysis, which is intended to generate hypotheses for testing in a future analysis on a larger dataset.

## Data Availability

The raw data supporting the conclusions of this article will be made available by the authors, without undue reservation.

## References

[B1] Adamson M. Main K. Milazzo A. Soman S. Kong J. Kolakowsky-Hayner S. (2020). Cortical thickness and diffusion properties in the injured brain: The influence of chronic health complaints. *Mil. Med.* 185 (Suppl. 1), 168–175. 10.1093/milmed/usz213 32074336

[B2] Alzheimer’s Association (2014). 2014 Alzheimer’s disease facts and figures. *Alzheimers Dement.* 10 e47–e92. 10.1016/j.jalz.2014.02.001 24818261

[B3] Amgalan A. Maher A. Ghosh S. Chui H. Bogdan P. Irimia A. (2022). Brain age estimation reveals older adults’ accelerated senescence after traumatic brain injury. *Geroscience* 44 2509–2525. 10.1007/s11357-022-00597-1 35792961 PMC9768106

[B4] Ashburner J. Barnes G. Chen C. Daunizeau J. Flandin G. Friston K. (2014). *SPM12 Manual.* London: Wellcome Trust Cent Neuroimaging.

[B5] Bai B. Vanderwall D. Li Y. Wang X. Poudel S. Wang H. (2021). Proteomic landscape of Alzheimer’s Disease: Novel insights into pathogenesis and biomarker discovery. *Mol. Neurodegener.* 16:55. 10.1186/s13024-021-00474-z 34384464 PMC8359598

[B6] Benjamini Y. Hochberg Y. (1995). Controlling the false discovery rate: A practical and powerful approach to multiple testing. *J. R. Stat. Soc. Ser. B Methodol.* 57 289–300. 10.1111/j.2517-6161.1995.tb02031.x

[B7] Blevins C. Weathers F. Davis M. Witte T. Domino J. (2015). The Posttraumatic stress disorder checklist for DSM-5 (PCL-5): Development and initial psychometric evaluation. *J. Trauma. Stress* 28 489–498. 10.1002/jts.22059 26606250

[B8] Brasure M. Lamberty G. Sayer N. Nelson N. MacDonald R. Ouellette J. (2012). *Multidisciplinary Postacute Rehabilitation for Moderate to Severe Traumatic Brain Injury in Adults.* Rockville, MD: Agency for Healthcare Research and Quality (US). 22834016

[B9] Chen J. Lin K. Chen Y. (2009). Risk factors for dementia. *J. Formos Med. Assoc.* 108 754–764. 10.1016/S0929-6646(09)60402-2 19864195

[B10] Cheng G. Huang C. Deng H. Wang H. (2012). Diabetes as a risk factor for dementia and mild cognitive impairment: A meta-analysis of longitudinal studies. *Intern. Med. J.* 42 484–491. 10.1111/j.1445-5994.2012.02758.x 22372522

[B11] Cole J. (2018). *brainageR: Brain age Prediction in R.* London: King’s College London.

[B12] Cole J. Franke K. (2017). Predicting age using neuroimaging: Innovative brain ageing biomarkers. *Trends Neurosci.* 40 681–690. 10.1016/j.tins.2017.10.001 29074032

[B13] Cole J. Leech R. Sharp D. (2015). Alzheimer’s Disease neuroimaging initiative. Prediction of brain age suggests accelerated atrophy after traumatic brain injury. *Ann. Neurol.* 77 571–581. 10.1002/ana.24367 25623048 PMC4403966

[B14] Cole J. Marioni R. Harris S. Deary I. (2019). Brain age and other bodily ‘ages’: Implications for neuropsychiatry. *Mol. Psychiatry* 24 266–281. 10.1038/s41380-018-0098-1 29892055 PMC6344374

[B15] Cole J. Poudel R. Tsagkrasoulis D. Caan M. Steves C. Spector T. (2017). Predicting brain age with deep learning from raw imaging data results in a reliable and heritable biomarker. *Neuroimage* 163 115–124. 10.1016/j.neuroimage.2017.07.059 28765056

[B16] Cole J. Ritchie S. Bastin M. Valdés Hernández M. Muñoz Maniega S. Royle N. (2018). Brain age predicts mortality. *Mol. Psychiatry* 23 1385–1392. 10.1038/mp.2017.62 28439103 PMC5984097

[B17] Collins J. Woodhouse A. Bye N. Vickers J. King A. Ziebell J. (2020). Pathological links between traumatic brain injury and dementia: Australian pre-clinical research. *J. Neurotrauma.* 37 782–791. 10.1089/neu.2019.6906 32046575

[B18] Cunningham J. Johnson R. Litzelman K. Skinner H. Seo S. Engelman C. (2013). Telomere length varies by DNA extraction method: Implications for epidemiologic research. *Cancer Epidemiol. Biomarkers Prev.* 22 2047–2054. 10.1158/1055-9965.EPI-13-0409 24019396 PMC3827976

[B19] Dams-O’Connor K. Gibbons L. Bowen J. McCurry S. Larson E. Crane P. (2013). Risk for late-life re-injury, dementia and death among individuals with traumatic brain injury: A population-based study. *J. Neurol Neurosurg. Psychiatry* 84 177–182. 10.1136/jnnp-2012-303938 23172868 PMC3752841

[B20] Dennis E. Taylor B. Newsome M. Troyanskaya M. Abildskov T. Betts A. (2022). Advanced brain age in deployment-related traumatic brain injury: A LIMBIC-CENC neuroimaging study. *Brain Inj.* 36 662–672. 10.1080/02699052.2022.2033844 35125044 PMC9187589

[B21] Dennis E. Vervoordt S. Adamson M. Houshang A. Bigler E. Caeyenberghs K. (2024). Accelerated aging after traumatic brain injury: An ENIGMA multi-cohort mega-analysis. *Ann. Neurol.* 96 365–377. 10.1002/ana.26952 38845484 PMC13431743

[B22] Desikan R. Ségonne F. Fischl B. Quinn B. Dickerson B. Blacker D. (2006). An automated labeling system for subdividing the human cerebral cortex on MRI scans into gyral based regions of interest. *Neuroimage* 31 968–980. 10.1016/j.neuroimage.2006.01.021 16530430

[B23] Dewan M. Rattani A. Fieggen G. Arraez M. Servadei F. Boop F. (2019). Global neurosurgery: The current capacity and deficit in the provision of essential neurosurgical care. Executive summary of the global neurosurgery initiative at the program in global surgery and social change. *J. Neurosurg.* 130 1055–1064. 10.3171/2017.11.JNS171500 29701548

[B24] Ertekin T. Acer N. Içer S. Ilıca A. (2013). Comparison of two methods for the estimation of subcortical volume and asymmetry using magnetic resonance imaging: A methodological study. *Surg. Radiol. Anat.* 35 301–309. 10.1007/s00276-012-1036-6 23143016

[B25] Fernández-Calle R. Konings S. Frontiñán-Rubio J. García-Revilla J. Camprubí-Ferrer L. Svensson M. (2022). APOE in the bullseye of neurodegenerative diseases: impact of the APOE genotype in Alzheimer’s disease pathology and brain diseases. *Mol. Neurodegener.* 17:62. 10.1186/s13024-022-00566-4 36153580 PMC9509584

[B26] Fischl B. Sereno M. Dale A. (1999a). Cortical surface-based analysis. II: Inflation, flattening, and a surface-based coordinate system. *Neuroimage* 9 195–207. 10.1006/nimg.1998.0396 9931269

[B27] Fischl B. Sereno M. Tootell R. Dale A. (1999b). High-resolution intersubject averaging and a coordinate system for the cortical surface. *Hum. Brain Mapp.* 8 272–284. 10.1002/(sici)1097-019319998:4&272::aid-hbm10&3.0.co;2-4 10619420 PMC6873338

[B28] Fischl B. van der Kouwe A. Destrieux C. Halgren E. Ségonne F. Salat D. (2004). Automatically parcellating the human cerebral cortex. *Cereb. Cortex* 14 11–22. 10.1093/cercor/bhg087 14654453

[B29] Fleminger S. Oliver D. Lovestone S. Rabe-Hesketh S. Giora A. (2003). Head injury as a risk factor for Alzheimer’s disease: The evidence 10 years on; a partial replication. *J. Neurol. Neurosurg. Psychiatry* 74 857–862. 10.1136/jnnp.74.7.857 12810767 PMC1738550

[B30] Franke K. Gaser C. (2012). Longitudinal changes in individual brain AGE in healthy aging, mild cognitive impairment, and Alzheimer’s Disease. *GeroPsych* 25 235–245. 10.1024/1662-9647/a000074

[B31] Franke K. Gaser C. (2019). Ten years of brain AGE as a neuroimaging biomarker of brain aging: What insights have we gained? *Front. Neurol.* 10:789. 10.3389/fneur.2019.00789 31474922 PMC6702897

[B32] Franke K. Gaser C. Manor B. Novak V. (2013). Advanced brain AGE in older adults with type 2 diabetes mellitus. *Front. Aging Neurosci.* 5:90. 10.3389/fnagi.2013.00090 24381557 PMC3865444

[B33] Franke K. Ziegler G. Klöppel S. Gaser C. (2010). Estimating the age of healthy subjects from T1-weighted MRI scans using kernel methods: Exploring the influence of various parameters. *Neuroimage* 50 883–892. 10.1016/j.neuroimage.2010.01.005 20070949

[B34] Fujita S. Mori S. Onda K. Hanaoka S. Nomura Y. Nakao T. (2023). Characterization of brain volume changes in aging individuals with normal cognition using serial magnetic resonance imaging. *JAMA Netw. Open* 6:e2318153. 10.1001/jamanetworkopen.2023.18153 37378985 PMC10308250

[B35] Gaser C. Franke K. Klöppel S. Koutsouleris N. Sauer H. (2013). Brain AGE in mild cognitive impaired patients: Predicting the conversion to Alzheimer’s Disease. *PLoS One* 8:e67346. 10.1371/journal.pone.0067346 23826273 PMC3695013

[B36] Hedderich D. Menegaux A. Schmitz-Koep B. Nuttall R. Zimmermann J. Schneider S. (2022). Increased brain age gap estimate (BrainAGE) in young adults after premature birth. *Front. Aging Neurosci.* 13:653365. 10.3389/fnagi.2021.653365 33867970 PMC8047054

[B37] Jirsaraie R. Gorelik A. Gatavins M. Engemann D. Bogdan R. Barch D. (2023). A systematic review of multimodal brain age studies: Uncovering a divergence between model accuracy and utility. *Patterns* 4:100712. 10.1016/j.patter.2023.100712 37123443 PMC10140612

[B38] Kang X. Yund E. Herron T. Woods D. (2007). Improving the resolution of functional brain imaging: Analyzing functional data in anatomical space. *Magn. Reson. Imaging* 25 1070–1078. 10.1016/j.mri.2006.12.005 17707169

[B39] Karatzoglou A. Smola A. Hornik K. Zeileis A. (2004). kernlab - An S4 package for kernel methods in R. *J. Stat. Softw.* 11 1–20. 10.18637/jss.v011.i09

[B40] Kaufmann T. van der Meer D. Doan N. Schwarz E. Lund M. Agartz I. (2019). Common brain disorders are associated with heritable patterns of apparent aging of the brain. *Nat. Neurosci.* 22 1617–1623. 10.1038/s41593-019-0471-7 31551603 PMC6823048

[B41] Kolenic M. Franke K. Hlinka J. Matejka M. Capkova J. Pausova Z. (2018). Obesity, dyslipidemia and brain age in first-episode psychosis. *J. Psychiatr. Res.* 99 151–158. 10.1016/j.jpsychires.2018.02.012 29454222

[B42] Korbmacher M. de Lange A. van der Meer D. Beck D. Eikefjord E. Lundervold A. (2023a). Brain-wide associations between white matter and age highlight the role of fornix microstructure in brain ageing. *Hum. Brain Mapp.* 44 4101–4119. 10.1002/hbm.26333 37195079 PMC10258541

[B43] Korbmacher M. Gurholt T. de Lange A. van der Meer D. Beck D. Eikefjord E. (2023b). Bio-psycho-social factors’ associations with brain age: A large-scale UK Biobank diffusion study of 35,749 participants. *Front. Psychol.* 14:1117732. 10.3389/fpsyg.2023.1117732 37359862 PMC10288151

[B44] Korbmacher M. van der Meer D. Beck D. Askeland-Gjerde D. Eikefjord E. Lundervold A. (2024a). Distinct longitudinal brain white matter microstructure changes and associated polygenic risk of common psychiatric disorders and Alzheimer’s disease in the UK Biobank. *Biol. Psychiatry Glob. Open Sci.* 4:100323. 10.1016/j.bpsgos.2024.100323 39132576 PMC11313202

[B45] Korbmacher M. van der Meer D. Beck D. de Lange A. Eikefjord E. Lundervold A. (2024b). Brain asymmetries from mid- to late life and hemispheric brain age. *Nat. Commun.* 15:956. 10.1038/s41467-024-45282-3 38302499 PMC10834516

[B46] Korbmacher M. Vidal-Pineiro D. Wang M. van der Meer D. Wolfers T. Nakua H. (2024c). Cross-sectional brain age assessments are limited in predicting future brain change. *Hum. Brain Mapp.* 46:e70203. 10.1002/hbm.70203 40235434 PMC12000824

[B47] Korbmacher M. Wang M. Eikeland R. Buchert R. Andreassen O. Espeseth T. (2023c). Considerations on brain age predictions from repeatedly sampled data across time. *Brain Behav.* 13:e3219. 10.1002/brb3.3219 37587620 PMC10570486

[B48] Korbmacher M. Westlye L. Maximov I. (2024d). FreeSurfer version-shuffling can enhance brain age predictions. *NeuroImage Rep.* 4:100214. 10.1016/j.ynirp.2024.100214 PMC12172796 40568570

[B49] Kuiper J. Zuidersma M. Oude Voshaar R. Zuidema S. van den Heuvel E. Stolk R. (2015). Social relationships and risk of dementia: A systematic review and meta-analysis of longitudinal cohort studies. *Ageing Res. Rev.* 22 39–57. 10.1016/j.arr.2015.04.006 25956016

[B50] Leonardsen E. Peng H. Kaufmann T. Agartz I. Andreassen O. Celius E. (2022). Deep neural networks learn general and clinically relevant representations of the ageing brain. *Neuroimage* 256:119210. 10.1016/j.neuroimage.2022.119210 35462035 PMC7614754

[B51] Li Y. Li Y. Li X. Zhang S. Zhao J. Zhu X. (2017). Head injury as a risk factor for dementia and Alzheimer’s Disease: A systematic review and meta-analysis of 32 observational studies. *PLoS One* 12:e0169650. 10.1371/journal.pone.0169650 28068405 PMC5221805

[B52] Luders E. Cherbuin N. Gaser C. (2016). Estimating brain age using high-resolution pattern recognition: Younger brains in long-term meditation practitioners. *Neuroimage* 134 508–513. 10.1016/j.neuroimage.2016.04.007 27079530

[B53] Maas A. Menon D. Adelson P. Andelic N. Bell M. Belli A. (2017). Traumatic brain injury: Integrated approaches to improve prevention, clinical care, and research. *Lancet Neurol.* 16 987–1048. 10.1016/S1474-4422(17)30371-X 29122524

[B54] Martin-Ruiz C. Baird D. Roger L. Boukamp P. Krunic D. Cawthon R. (2015). Reproducibility of telomere length assessment: An international collaborative study. *Int. J. Epidemiol.* 44 1673–1683. 10.1093/ije/dyu191 25239152 PMC4681105

[B55] McInnes K. Friesen C. MacKenzie D. Westwood D. Boe S. (2017). Mild Traumatic Brain Injury (mTBI) and chronic cognitive impairment: A scoping review. *PLoS One* 12:e0174847. 10.1371/journal.pone.0174847 28399158 PMC5388340

[B56] McMillan T. Teasdale G. Weir C. Stewart E. (2011). Death after head injury: The 13 year outcome of a case control study. *J. Neurol. Neurosurg. Psychiatry* 82 931–935. 10.1136/jnnp.2010.222232 21282727

[B57] Mendez M. (2017). What is the relationship of traumatic brain injury to Dementia? *J. Alzheimers Dis.* 57 667–681. 10.3233/JAD-161002 28269777

[B58] Mollayeva T. Mollayeva S. Colantonio A. (2018). Traumatic brain injury: Sex, gender and intersecting vulnerabilities. *Nat. Rev. Neurol.* 14 711–722. 10.1038/s41582-018-0091-y 30397256

[B59] More S. Antonopoulos G. Hoffstaedter F. Caspers J. Eickhoff S. Patil K. (2023). Brain-age prediction: A systematic comparison of machine learning workflows. *Neuroimage* 270:119947. 10.1016/j.neuroimage.2023.119947 36801372

[B60] Nenadić I. Hoof A. Dietzek M. Langbein K. Reichenbach J. Sauer H. (2017). Diffusion tensor imaging of cingulum bundle and corpus callosum in schizophrenia vs. bipolar disorder. *Psychiatry Res. Neuroimaging* 266 96–100. 10.1016/j.pscychresns.2017.05.011 28644999

[B61] Nichols E. Steinmetz J. Vollset S. Fukutaki K. Chalek J. Abd-Allah F. (2022). Estimation of the global prevalence of dementia in 2019 and forecasted prevalence in 2050: An analysis for the Global Burden of Disease Study 2019. *Lancet Public Health* 7 e105–e125. 10.1016/S2468-2667(21)00249-8 34998485 PMC8810394

[B62] Parker J. Cutler C. Heaslip V. (2020). Dementia as zeitgeist: Social problem construction and the role of a contemporary distraction. *Sociol. Res. Online* 26:1360780420929033. 10.1177/1360780420929033

[B63] Prince M. Bryce R. Albanese E. Wimo A. Ribeiro W. Ferri C. (2013). The global prevalence of dementia: A systematic review and meta analysis. *Alzheimers Dement.* 9 63–75.e2. 10.1016/j.jalz.2012.11.007 23305823

[B64] Roebuck-Spencer T. Cernich A. (2014). “Epidemiology and societal impact of traumatic brain injury,” in *Handbook on the Neuropsychology of Traumatic Brain Injury*, eds Sherer M. Sander A. (New York, NY: Springer), 3–23. 10.1007/978-1-4939-0784-7_1

[B65] Rogenmoser L. Kernbach J. Schlaug G. Gaser C. (2018). Keeping brains young with making music. *Brain Struct. Funct.* 223 297–305. 10.1007/s00429-017-1491-2 28815301

[B66] Rokicki J. Wolfers T. Nordhøy W. Tesli N. Quintana D. Alnaes D. (2021). Multimodal imaging improves brain age prediction and reveals distinct abnormalities in patients with psychiatric and neurological disorders. *Hum. Brain Mapp.* 42 1714–1726. 10.1002/hbm.25323 33340180 PMC7978139

[B67] Sanders J. Newman A. (2013). Telomere length in epidemiology: A biomarker of aging, age-related disease, both, or neither? *Epidemiol. Rev.* 35 112–131. 10.1093/epirev/mxs008 23302541 PMC4707879

[B68] Schrouff J. Rosa M. Rondina J. Marquand A. Chu C. Ashburner J. (2013). PRoNTo: Pattern recognition for neuroimaging toolbox. *Neuroinformatics* 11 319–337. 10.1007/s12021-013-9178-1 23417655 PMC3722452

[B69] Schwarz C. Gunter J. Wiste H. Przybelski S. Weigand S. Ward C. (2016). A large-scale comparison of cortical thickness and volume methods for measuring Alzheimer’s disease severity. *Neuroimage Clin.* 11 802–812. 10.1016/j.nicl.2016.05.017 28050342 PMC5187496

[B70] Sled J. Zijdenbos A. Evans A. C. (1998). A nonparametric method for automatic correction of intensity nonuniformity in MRI data. *IEEE Trans. Med. Imaging* 17 87–97. 10.1109/42.668698 9617910

[B71] Smits L. van Harten A. Pijnenburg Y. Koedam E. Bouwman F. Sistermans N. (2015). Trajectories of cognitive decline in different types of dementia. *Psychol. Med.* 45 1051–1059. 10.1017/S0033291714002153 25229325

[B72] Spitz G. Hicks A. Roberts C. Rowe C. Ponsford J. (2022). Brain age in chronic traumatic brain injury. *Neuroimage Clin.* 35:103039. 10.1016/j.nicl.2022.103039 35580421 PMC9117693

[B73] Tetereva A. Pat N. (2023). *The (Limited?) Utility of Brain Age as a Biomarker for Capturing Fluid Cognition in Older Individuals.* New Zealand: University of Otago. 10.7554/eLife.87297 PMC11175613 38869938

[B74] Thambisetty M. Wan J. Carass A. An Y. Prince J. Resnick S. (2010). Longitudinal changes in cortical thickness associated with normal aging. *Neuroimage* 52 1215–1223. 10.1016/j.neuroimage.2010.04.258 20441796 PMC2910226

[B75] Thurman D. Alverson C. Dunn K. Guerrero J. Sniezek J. (1999). Traumatic brain injury in the United States: A public health perspective. *J. Head Trauma. Rehabil.* 14 602–615. 10.1097/00001199-199912000-00009 10671706

[B76] Vidal-Pineiro D. Wang Y. Krogsrud S. Amlien I. Baaré W. Bartres-Faz D. (2021). Individual variations in ‘brain age’ relate to early-life factors more than to longitudinal brain change. *Elife* 10:e69995. 10.7554/eLife.69995 34756163 PMC8580481

[B77] Wang M. Korbmacher M. Eikeland R. Nerland S. Vidal-Pineiro D. Specht K. (2024). The within-subject stability of cortical thickness, surface area, and brain volumes across one year. *bioRxiv [Preprint]* 10.1101/2024.06.01.596956

[B78] Wolters F. Segufa R. Darweesh S. Bos D. Ikram M. Sabayan B. (2018). Coronary heart disease, heart failure, and the risk of dementia: A systematic review and meta-analysis. *Alzheimers Dement.* 14 1493–1504. 10.1016/j.jalz.2018.01.007 29494808

[B79] World Health Organization [WHO] (2021). *Dementia.* Available online at: https://www.who.int/news-room/fact-sheets/detail/dementia (accessed May 28, 2021).

